# Geminivirus mixed infection on pepper plants: Synergistic interaction between PHYVV and PepGMV

**DOI:** 10.1186/1743-422X-8-104

**Published:** 2011-03-08

**Authors:** Ilenia Rentería-Canett, Beatriz Xoconostle-Cázares, Roberto Ruiz-Medrano, Rafael F Rivera-Bustamante

**Affiliations:** 1 Departamento de Ingeniería Genética. Centro de Investigación y de Estudios Avanzados del IPN (Cinvestav), Unidad Irapuato, Km. 9.6 Libramiento Norte, 36821 Irapuato, Guanajuato; 2 Departamento de Biotecnología y Bioingeniería, Cinvestav-IPN, Av. IPN 2508, San Pedro Zacatenco, 07360 México, DF

## Abstract

**Background:**

PHYVV and PepGMV are plant viruses reported in Mexico and Southern US as causal agents of an important pepper disease known as "rizado amarillo". Mixed infections with PHYVV and PepGMV have been reported in several hosts over a wide geographic area. Previous work suggested that these viruses might interact at the replication and/or movement level in a complex manner. The aim of present report was to study some aspects of a synergistic interaction between PHYVV and PepGMV in pepper plants. These include analyses of symptom severity, viral DNA concentration and tissue localization of both viruses in single and mixed infections.

**Results:**

Mixed infections with PepGMV and PHYVV induced symptoms more severe than those observed in single viral infections. Whereas plants infected with either virus (single infection) presented a remission stage with a corresponding decrease in viral DNA levels, double-infected plants did not present symptom remission and both viral DNA concentrations dramatically increased. *In situ *hybridization experiments revealed that both viruses are restricted to the vascular tissue. Interestingly, the amount of viral DNA detected was higher in plants inoculated with PepGMV than that observed in PHYVV-infected plants. During mixed infections, the location of both viruses remained similar to the one observed in single infections, although the number of infected cells increases. Infections with the tripartite mixture PHYVV (A+B) + PepGMV A produced a similar synergistic infection to the one observed after inoculation with both full viruses. On the contrary, tripartite mixture PepGMV (A+B) + PHYVV A did not produce a synergistic interaction. In an attempt to study the contribution of individual genes to the synergism, several mutants of PHYVV or PepGMV were inoculated in combination with the corresponding wild type, second virus (wt PepGMV or wt PHYVV). All combinations tested resulted in synergistic infections, with exception of the TrAP mutant of PepGMV (PepGMV TrAP-) + PHYVV.

**Conclusion:**

In this report, we have demonstrated that synergistic interaction between PHYVV and PepGMV during a mixed infection is mainly due to an increased DNA concentration of both viruses, without any noticeable effect on the localization of either virus on infected plant tissue. Our results have shown that the viral component A from PepGMV is important for synergism during PHYVV-PepGMV mixed infections.

## Background

**Figure 1:**
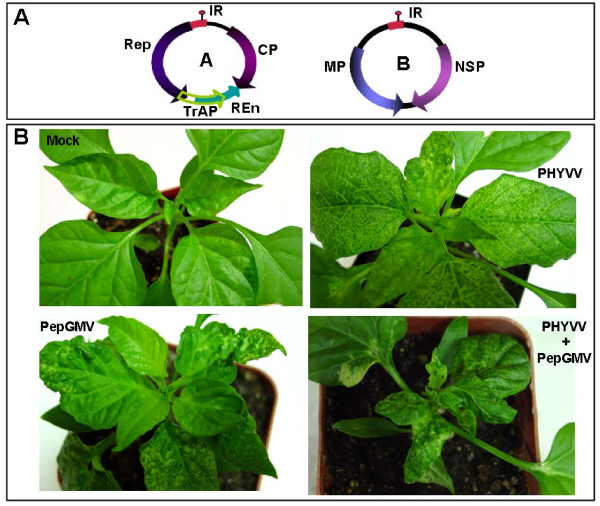
**Begomovirus genome organization and symptom characterization following inoculation of pepper plants with single or mixed viruses**. **Panel A**, Genomic map showing: A Component (CP, coat protein; Rep, viral replication-associated protein; TrAP, transcriptional activator protein; REn, replication enhancement protein), B Component (MP, movement protein and NSP, nuclear-shuttle protein) and intergenic region (IR). **Panel B**, symptoms following inoculation of pepper plants 21 days postinoculation (dpi): Mock, (inoculated plant with pBluescriptSK(+) plasmid); PHYVV, plant inoculated with PHYVV going through symptom recovery (remission); PepGMV, plant inoculated with PepGMV showing remission; PHYVV + PepGMV, plant inoculated with PHYVV and PepGMV displaying synergism.

Geminiviruses are plant viruses that have a wide host range. They possess a circular, single-stranded DNA (ssDNA) genome packaged into isometric twinned particles. Based on genome organization, host range and insect vector, geminiviruses are divided into four genera: *Mastrevirus*, *Curtovirus*, *Topocuvirus *and *Begomovirus *[1]. *Pepper huasteco yellow vein virus *(PHYVV) and *Pepper golden mosaic virus *(PepGMV) are members of the genus *Begomovirus*. The genomes of these whitefly-transmitted viruses are organized into two circular ssDNA components, DNAs A and B (Figure 1A). The A component encodes four proteins: CP, coat protein; Rep, replication-associated protein; TrAP, transcription activator protein and REn, replication enhancer. The B component, encodes two movement proteins, the nuclear shuttle protein (NSP) and the movement protein (MP) [2,3]. PHYVV and PepGMV have been reported in Mexico and Southern United States as important viral pathogens in many economically important horticultural crops such as pepper, tomato, tomatillo (husk tomato), as well as tobacco. Several reports have demonstrated that mixed infections with PHYVV and PepGMV are common in those crops and, in many cases, this complex has become the predominant mixture [2,4-8].

The wide distribution in nature, both geographically and in terms of host range, presents the mixture PHYVV-PepGMV as an interesting, natural model for virus-virus interactions. Initial studies in several hosts (pepper, tobacco and *Nicotiana benthamiana*) have suggested that the interactions can occur at different levels (symptom severity, virus gene expression, replication and movement) and many additional factors such as type and age of host, order of virus arrival can also affect the outcome of the interaction [2,8]. It has been shown that mixed infections are a potential source of geminivirus variability due to recombination events. Some of the best studied examples include geminiviruses infecting cassava in Africa [9-12], and some related geminiviruses affecting bean/tomato [13,14]. In addition to be a source of geminivirus variability, mixed infections can also have a profound effect in productivity since they often result in synergistic interactions such as the ones reported with cassava geminiviruses in Africa [12,15], and tomato geminiviruses in several countries [16-18].

The mechanisms acting in the synergistic interaction are not well understood yet. It has been suggested that the silencing suppressor properties of some geminivirus proteins play an important role. Nevertheless, it has been also suggested that the silencing suppressor activity may vary from virus to virus and it could even reside in different proteins (e.g. TrAP or AC4) [15]. More recently, Alves *et al*. showed that virus interactions are complex processes that could include a negative interference (at certain stages/conditions) between the viruses involved, as well as synergism in other stages. This last case could result in a delocalization of a normally phloem-limited virus into mesophyll tissues [18].

Similar complexity has been observed in the case of the interaction between PHYVV and PepGMV as reported earlier [8]. In this report, we focused on the study of the location and replication of PHYVV and PepGMV during infection of pepper plants (*Capsicum annuum *L.) during both, single and mixed infections. Likewise, some experiments were performed in an attempt to elucidate which viral genes are important for the synergistic interaction observed between PHYVV-PepGMV in their natural host.

## Results

### Symptoms of pepper plants in single and mixed infections

Infection of pepper plants with PepGMV induces typical yellow mosaic and wrinkle symptoms in the two pairs of leaves that emerged between 7 to 14 days post inoculation (dpi). By day 21, the newly emerged leaves showed a reduction in severity of symptoms, a symptom remission process described earlier [19,20]. Similar results are observed in pepper plants infected with PHYVV. However, in this case the symptoms induced are usually milder than the ones induced by PepGMV and consist of vein yellowing as well as leaf curling [5]. In contrast, plants inoculated with the mixture PepGMV and PHYVV show more severe symptoms than those observed in either single infection (Figure 1B). In addition, mixed infected plants do not show remission and are usually unproductive.

### Viral DNA levels increase in mixed infection

**Figure 2:**
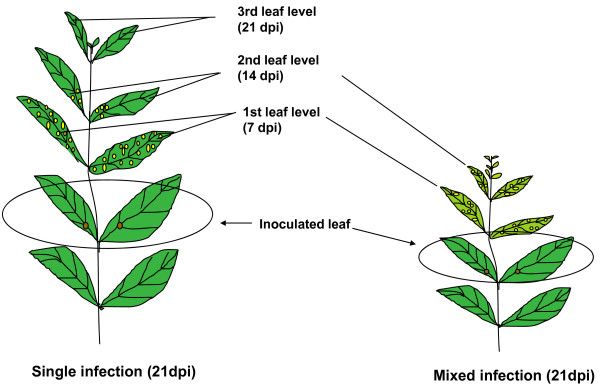
**Schematic representation of pepper plants inoculated with one or two viruses**. The figure indicates the inoculated leaf as well as collected leaves for analysis.

**Figure 3:**
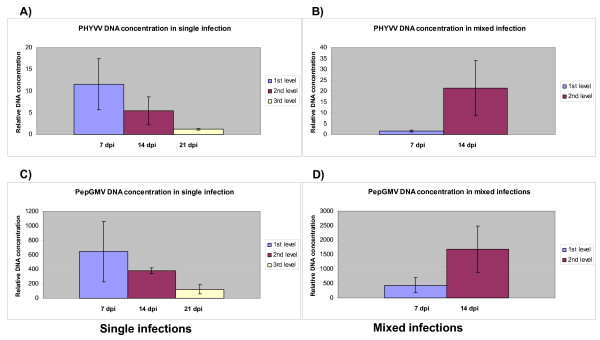
**Estimation of relative viral DNA levels in single and mixed infections on pepper plants**. Graphic representation of relative viral DNA levels from single and mixed infections performed by real-time PCR at indicated leaves level. **A) **PHYVV DNA levels in single infections, relative to days post-infection; **C) **PepGMV DNA levels in single infections, relative to days post-infection. A dramatic decrease of viral DNA levels on the third leaves level post inoculation is observed in single infections treatments. **B) **PHYVV DNA levels in mixed infections; **D) **PepGMV DNA levels in mixed infections. In this case these are increased of DNA viral in the second level leaves. Each bar represents mean values of five plants; minimum value of each component was taken as 1. Error bars plotted refer to standard errors of the mean.

An important parameter to be evaluated in synergistic interactions between viruses is the concentration of viral nucleic acids. For our purposes, the level of both viral genomic DNAs was determined by real-time PCR assays. In the case of single-infected plants, apical leaf tissue was collected at 7, 14, 21 dpi, which represent the first three sets of leaves (stages) that appear after inoculation (Figure 2). Total DNA was extracted and viral DNA quantified by real time PCR. In the case of doubly infected plants, we were able to collect only the first and second set of leaves (7 and 14 dpi), since the symptoms were typically so severe by 21 dpi that it was practically impossible to distinguish and separate the new leaves to provide enough, equivalent material for the analysis (Figures 1B and 2). The plants remained severely stunted and unproductive. For the DNA quantification analysis, the concentration of both A components (PHYVV and/or PepGMV) was determined using the sets of primers described in Materials and Methods. To compare the amount of viral DNA in each tissue, all values were normalized using the results of a parallel quantification analysis of a host gene. As expected from previously reported results, the concentration of PHYVV DNA showed an initial high value in the first stage leaves (7 dpi). Viral DNA concentration, however, showed significant reduction in the following emerging leaves (second and third stages) that were concomitant with the reduction of symptoms severity (Figure 3A). Similar results were obtained with PepGMV infected plants (Figure 3C). In other words, a reduction of viral DNA corresponded with the recovery process as previously characterized [19,20]. Nevertheless, an important difference between PHYVV and PepGMV single infections was the level of viral DNA. Typically, the levels of PepGMV DNA in single infections were consistently higher (90 to 100 times) than the levels obtained for PHYVV. These differences were consistent in several experiments where independently infected plants were grown side by side in the same growth chamber (Figure 3A, C).

When both viral DNAs were analyzed in mixed infections, several interesting results were observed. First, the concentration of viral DNA obtained in the tissue collected at 7 dpi (first stage) was comparable in each case (PHYVV or PepGMV) to the concentrations obtained with he respective singly-infected plants (Figure 3). However, the concentration of both viral DNAs was dramatically increased in the second stage leaves (apical tissue collected at 14 dpi). Again, the levels of PepGMV DNA were consistently two orders of magnitude higher than levels from PHYVV DNA in mixed infections. The increase of both viral DNA concentrations in the mixed infection correlates with a typical synergistic interaction.

### Localization of PHYVV and PepGMV in infected pepper plants

**Figure 4:**
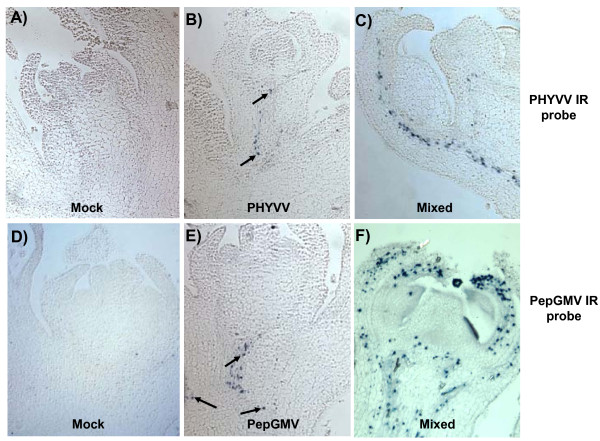
***In-situ *hybridization for location of PHYVV and PepGMV on pepper plants tissues**. A-C show hybridization with PHYVV intergenic region (IR) probe. **A) **mock-inoculated plant; **B) **PHYVV infected plant; **C) **plant with mixed infection. D-F show hybridization with PepGMV IR probe; **D) **mock-inoculated plant, **E) **plant infected with PepGMV, **F) **plant with mixed infection. In all cases, tissues from apical meristems were collected 14 dpi, and hybridized with digoxigenin-labeled probes with IR from either PHYVV or PepGMV.

Many begomoviruses have been reported as phloem-limited; however, in some other cases, virus particles/DNA have also been detected in other tissues, such as mesophyll cells. Some genetic components important for tissue specificity have been reported [21-23]. More recently, it has been suggested that tissue tropism may be altered as a result of a mixed infection [18]. It was of interest to determine the tissue tropism of both PepGMV and PHYVV, during single and mixed infection in a natural host. Therefore, *in situ *hybridization assays were carried out in systemically infected pepper plants. In PHYVV single infections, we found that this virus is restricted to vascular tissue and the number of infected cells is rather low (Figure 4B). Similarly, PepGMV in a single infection was also confined to vascular tissue (Figure 4E), however, the number of infected cells appears to be higher than the one observed for PHYVV-infected plants (Figure 4B and 4E).

**Figure 5:**
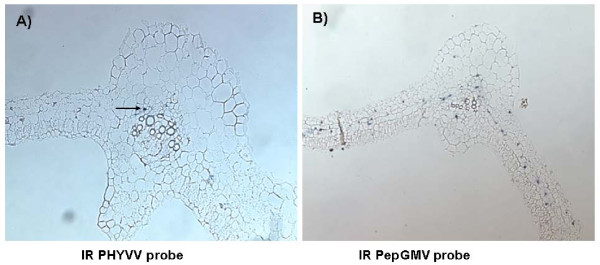
***In-situ *hybridization for location of PHYVV and PepGMV viruses on leaves tissues during mixed infections**. A and B, are sections of leaf with mixed infection. **A)**, hybridization with PHYVV intergenic region (IR) probe. **B)**, hybridization with PepGMV IR probe. The tissues from leaves were collected 14 dpi, and hybridized with digoxigenin-labeled probes with IR from either PHYVV or PepGMV.

In mixed infections, we found that both PHYVV and PepGMV remain restricted to vascular tissue on apical sections and leaves, i.e. no virus delocalization was observed (Figure 4C and 4F, Figure 5A and 5B). Nevertheless, the number of infected cells observed in these mixed infected tissues was higher than those founded in the corresponding single-infected plants (Figure 4B, C, E and 4F). Again, the number of PepGMV infected cells on the double inoculated plants (apex and leaf tissues) was consistently higher than the one observed for PHYVV (Figure 4F and 4C, Figure 5A and 5B). These results show that both viruses are restricted to vascular tissue in both types of infections (Figure 4B, C, E and 4F; Figure 5A and 5B). In addition, the results also suggest that the increase in the number of infected cells observed in mixed infections is, at least partially, responsible for the increase in viral DNA concentration.

### The mixture PHYVV + PepGMV A, but not PepGMV + PHYVV A, is sufficient for synergistic interaction

It was of interest to determine whether both genomes (PHVYVV and PepGMV) were necessary to generate a synergistic response. Therefore, plants were inoculated tripartite mixtures containing a full viral genome and the A component of the second virus. It was previously demonstrated that the component A of a given virus (either PHYVV or PepGMV) could not support the replication of the opposite B component [8].

**Figure 6:**
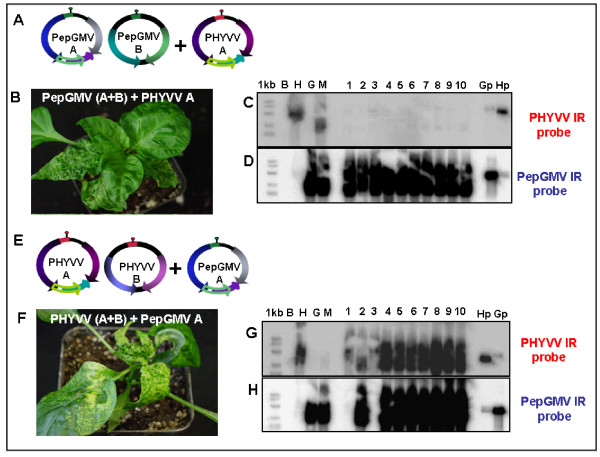
**Inoculation of pepper plants with tripartite mixtures**. **A) **and **E)**, schematic representations of components of tripartite mixtures used for inoculation; **B)**, symptoms observed on plants infected with the tripartite mixture PepGMV (A+B) + PHYVV A; **C) **and **D)**, Southern blot of DNA extracted from plants infected with tripartite mixture PepGMV (A+B) + PHYVV A; **F)**, symptoms observed on plants infected with tripartite mixture PHYVV (A+B) + PepGMV A; **G) **and **H)**, Southern blot of DNA extracted from plants infected with tripartite mixture PHYVV (A+B) + PepGMV A. The viral probe used in Southern blot is indicated. Southern blot lanes: 1 kb, molecular weight marker. B, mock-inoculated plant; H, PHYVV-infected plant; G, PepGMV-infected plant; M, plant infected with PHYVV + PepGMV mixture. Lanes 1 to 10 correspond to plants infected with tripartite mixture. Lane Gp corresponds to the cloned PepGMV genome; lane Hp, cloned PHYVV genome. Southern blot membranes were initially hybridized with PHYVV IR probe and later with PepGMV IR probe, after being regenerated.

After inoculation with different tripartite mixtures, plants were monitored for symptom expression to determine which plants had developed synergism. The mixture with PHYVV plus PepGMV A produces a synergistic interaction and plants infected with this combination never exhibited remission (Figure 6F; Table 1). It was possible to detect, in plants inoculated with this mixture, the replicative forms (RF, dsDNA) from both components of PHYVV, as well as the RF of PepGMV A (Figure 6G-H). In both cases, the concentration of viral DNA was higher than the one obtained in single-infected plants.

**Table 1:** Infectivity, symptoms and viral detection in peppers plants inoculated with PHYVV or/and PepGMV.

Inoculum	Infected plants^a^	Southern detection	Complementation	Remission	Synergism	Symptom severity †
PHYVV	20/20	√	na	√	Na	+ +

PepGMV	20/20	√	na	√	na	+ + +

PHYVV + PepGMV	20/20	Both viruses √	na	X	√	+ + + + +

PHYVV + PepGMV A	20/20	Both viruses √	√	X	√	+ + + + +

PepGMV + PHYVV A	20/20	Only PepGMV √	X	√	X	+ + +

PHYVV Rep-	0/20	X	na	na	na	-

PHYVV Rep- + PepGMV	20/20	Only PepGMV √	X	√	X	+ + +

PepGMV Rep-	0/20	X	na	na	na	-

PepGMV Rep- + PHYVV	20/20	Only PHYVV √	X	√	X	+ +

PHYVV TrAP-	0/20	X	na	na	na	-

PHYVV TrAP- + PepGMV	20/20	Both viruses √	√	√	√	+ + + +

PepGMV TrAP-	0/20	X	na	na	na	-

PepGMV TrAP- + PHYVV	20/20		X	√	X	+ +

PHYVV CP-	20/20**^b^**	√	na	√	na	+

PHYVV CP- + PepGMV	20/20	Both viruses √	√	√	√	+ + + +

PepGMV CP-	20/20	√	na	√	na	+

PepGMV CP- + PHYVV	20/20	Both viruses √	√	√	√	+ + + +

**^a ^**Number of infected plants/number of inoculated plants

**^b ^**Only detected on inoculated leaves

**† **Symptom severity was scored from mild (+) to severe (+++++)

**na: **not applicable, √: Yes, X: No

On the contrary, the tripartite mixture of PepGMV in combination with PHYVV A did not exhibit synergism and the infected plants did develop remission or symptom recovery (Figure 6B; Table 1). In plants inoculated with this tripartite mixture, only the replicative forms of PepGMV were detected in systemic tissue (Figure 6D; Table 1).

These results indicate that the combination of PepGMV A and PHYVV is sufficient for the establishment of a viral synergistic interaction in pepper plants. The reciprocal mixture failed to establish a synergistic infection, probably due to the lack the replication and systemic movement of PHYVV A as previously reported [8].

### Coat proteins are dispensable for a synergistic interaction

To verify if a specific viral gene is necessary or required to produce a synergistic interaction, several experiments using viral mutants were carried out. Table 1 shows the mutants used in the inoculation experiments and summarizes the results obtained. All viral mutants were assayed in either single or mixed infections and compared with parallel inoculations using wild type viruses. After inoculation, the plants were observed for symptom development, recovery process and analyzed for presence of viral RF. PHYVV and PepGMV CP mutants, when inoculated as a single infection, usually produced mild symptoms in the inoculated leaves, and rarely in systemic tissues. Previously, it had been reported that CP mutants of PHYVV could move systemically in pepper and *Nicotiana benthamiana *but not in tobacco plants [24] demonstrating that CP is dispensable in some hosts. As previously reported, mutations in *Rep *and *TrAP *genes for both PHYVV and PepGMV are either lethal (*Rep*) or severely affect the virus cycle (*TrAP*) that practically renders them as non-infective since no symptoms were induced, and viral DNA was barely detectable in systemic tissue of a few inoculated plants using highly sensitive procedures such as PCR (above 30 cycles).

**Figure 7:**
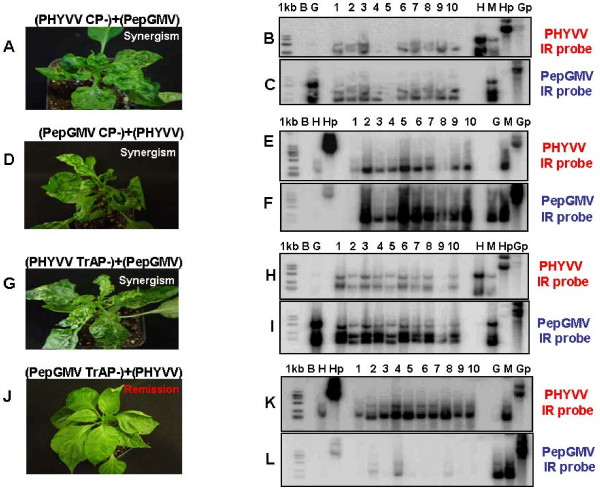
**Analysis of symptoms after infection with combinations of wild type and mutant viruses, and identification of replicative forms**. **A)**, Plant showing synergistic symptomatology due to mixed infection with PHYVV CP- + PepGMV; **B) **and **C)**, Southern blot of plants infected with PHYVV CP- + PepGMV; **D)**, Plant showing symptoms caused by viral synergism after mixed infection with PepGMV CP- + PHYVV; **E) **and **F)**, Southern blot of plants infected with PepGMV CP- + PHYVV; **G)**, Synergism after mixed infection with PHYVV TrAP- + PepGMV; **H) **and **I)**, Southern blot of plants infected with PHYVV TrAP- + PepGMV; **J)**, mixed infection with PepGMV TrAP- + PHYVV leading to symptom recovery of pepper; **K) **and **L)**, Southern blot of plants infected with PepGMV TrAP- + PHYVV. Southern blot lanes: B, mock-inoculated plant; H, PHYVV-infected plant; G, PepGMV-infected plant; M, plant infected with PHYVV + PepGMV mixture. Lanes 1 to 10, plants infected with mutants and wild type viruses. Gp lane, cloned PepGMV genome; Hp lane, cloned PHYVV genome. Membranes were initially hybridized with PHYVV IR probe and afterwards with PepGMV IR probe, after regeneration.

Nevertheless, some synergistic interactions were observed in plants inoculated with some PHYVV mutants in combination with wild type (wt) PepGMV or vice versa (Figure 7A, D and 7G, Table 1). Coat protein mutant from both viruses were still able to induce synergism when inoculated with the corresponding wt virus. The enhanced symptoms were similar to the ones obtained with both wt viruses. TrAP mutants, on the other hand, produced contrasting results. PHYVV TrAP-, by itself, was not infectious and no viral DNA was recovered from the inoculated plants. However, when inoculated with PepGMV, PHYVV TrAP-was able to replicate and induce a synergistic reaction. On the contrary, the combination of PHYVV and PepGMV TrAP-did not produce synergism. The infected plants showed mild, PHYVV-like symptoms and displayed a recovery as if infected only with PHYVV. When the infected plants with PHYVV and PepGMV TrAP mutant were analyzed for virus DNA, PepGMV TrAP-DNA was barely detected by Southern blot analysis in a low percentage of the inoculated plants (Figure 7L). These results suggest that PepGMV TrAP is able to complement PHYVV TrAP-whereas the inverse process (PHYVV complementing PepGMV TrAP-) is not productive. Additionally, these results also demonstrate that PHYVV TrAP is not required for synergism.

## Discussion

Mixed infections may result in additive, synergistic or antagonistic interactions. In the case of the synergistic interaction, disease complexes elicit symptoms that are more severe than the ones induced individually by the members of the complex. This is a fact of economical concern, and a very interesting biological question. Mixed infections also provide the opportunity for recombination between co-infecting viruses to give rise to new variants or species. Some of these new entities might become a severe phytopathological problem. Mixed viral infections in plants are quite common in nature. Most of the initial studies of plant-virus interaction were based on single infection models. Fortunately, the number of reports on mixed infection has increased recently generating information that now can be used to develop strategies to control complex diseases. The aim of this study was to continue the study of the interactions that occur between PHYVV and PepGMV, a natural mixture in many solanaceous crops, including pepper [2,8].

Virus concentrations in single and mixed infections were previously determined using Southern blot assays [8]. In this work, DNA concentration was determined by real-time PCR, a procedure that allows a more direct and accurate quantification of DNA concentration. From the viral DNA quantification experiments, several conclusions could be drawn. First, as reported before, viral DNA concentration in single infections decreases over time, and in a parallel manner to the recovery process previously reported [19,20]. Second, the concentration of PepGMV DNA is 10 fold higher than that of PHYVV in equivalent single-infected plants. This result was somewhat surprising considering that is one order of magnitude in concentration difference whereas the tissue tropism of both viruses is similar. Third, in mixed infected plants, as previously assessed by Southern blot analysis, both viral DNA concentrations increased over time since plants do not show recovery. Unfortunately, the mixture of viruses caused symptoms so severe (Figure 1) that apical growth practically stopped and it was no longer possible to dissect the apex at the third set-point (21 dpi) to recover independent leaves in an adequate manner to analyze and compare virus concentrations. Therefore, the results of the third sample were not reproducible. Finally, the enhanced PHYVV concentration in mixed infected plants never reached the levels observed with PepGMV, even in single infections.

It is not clear yet why two related viruses, with a similar host range and tissue tropism, will accumulate to such different concentration levels in a common host. A possibility is that PepGMV is more efficient than PHYVV in its replicative process accumulating to a higher level in each infected cell. However, our *in situ *hybridization experiments results suggest that the number of infected cells in the vascular tissue is at least partially responsible for the observed difference (PepGMV >> PYHVV). Another factor that might contribute to this difference is a differential fitness. PepGMV might have a better capability in dealing with host defenses to allow an efficient replication and movement process. In some RNA viruses, an efficient gene silencing suppressor activity has been shown to be important. In the case of geminiviruses, two proteins, TrAP and AC4 have been implicated in PTGS (see below). The fact that PHYVV accumulates to a higher level in double infections but it never reaches the levels observed in PepGMV, suggests that PHYVV (as any virus) possesses its own replicative regulation that is independent from the gene silencing mechanism. In other words, each virus seems to maintain its own regulatory mechanism that fine-tunes its independent replicative process.

In addition to determine the number of infected cells, the *in situ *hybridization experiments also provided information on the tissue tropism of each virus in both single and mixed infections. Tissue tropism of different geminiviruses has been reported in different hosts. Several monopartite (*Tomato leaf curl virus*, TLCV) and bipartite begomoviruses (*Abutilon mosaic virus*, AbMV; *Bean golden mosaic virus*, BGMV; *Indian cassava mosaic virus*, ICMV) have been reported as phloem-limited [25-28]. In some other cases, bipartite begomoviruses are able to infect palisade and spongy parenchyma as well as epidermal cells, for example *African cassava mosaic virus *(ACMV), *Bean dwarf mosaic virus *(BDMV) and *Tomato golden mosaic virus *(TGMV) [22,29]. Tissue tropism has been also investigated in geminiviral mixed infections. Morilla *et al*. reported that both, *Tomato yellow leaf curl virus *(TYLCV) and *Tomato yellow leaf curl Sardinia virus *(TYLCSV) were confined to the phloem in all tissues tested, in either single or mixed infections [16]. Other reports showed that in infections in *N. benthamiana*, *Tomato yellow spot virus *(ToYSV) is able to invade mesophyll cells, whereas the related begomovirus *Tomato rugose mosaic virus *(ToRMV) does not. However, in dual infections, ToRMV is no longer confined to the phloem and can be found in mesophyll cells similar to ToYSV [18], suggesting that ToYSV is able to facilitate the "escape" of ToRMV from the phloem and towards mesophyll tissue.

In the interaction reported here, both viruses were found restricted to the vascular tissue in both single and mixed infections. These results agreed with earlier observation using a low-resolution tissue printing technique. The vasculature restriction was also suggested in transgenic tobacco plants in which β-glucuronidase (*uidA *gene) was expressed under the direction of several viral gene promoters (i.e., *CP *and *Rep *genes) [30]. In the case of the mixed infections, we found no tissue delocalization in both viruses, only an increment in the number of infected cells. Since both viruses are restricted to the vasculature in single infections, it is not surprising that in the mixture they remain confined to that specific tissue. Nevertheless, it is an important confirmation since previous data with transgenic tobacco plants expressing *uidA *gene under the direction of several PHYVV and PepGMV promoters have showed a strong expression in tissues other than phloem. Our observations confirm that such type of results (transgenic plant expressing GUS with viral promoters) should be carefully analyzed since they do not necessarily reflect the activity of viral gene promoters in the context of an infection.

Previously, it was reported that the exchange of genomic components between PHYVV and PepGMV (PHYVV A + PepGMV B or PepGMV A + PHYVV B) did not generate infectious pseudorecombinant viruses [8]. In addition, it was reported that an asymmetric movement complementation occurred between these two viruses since PHYVV was able to move PepGMV A whereas the opposite mixture (PepGMV + PHYVV A) did not produce the same results. Thus, in this report we further explored possible synergistic effect of these tripartite mixtures. We found that, as expected from the previous results, the tripartite combination of PHYVV + PepGMV A produces synergism in pepper plants as observed with the enhanced symptoms and the increased DNA concentration levels (Figure 6). On the other hand, the combination of PepGMV and PHYVV A did not render synergism, perhaps due to the lack of complementation in movement as reported earlier. Synergism with tripartite mixtures has also been reported in other models systems [17]. However, the ability of those viruses *Tomato leaf curl New Delhi virus *(ToLCNDV) and *Tomato leaf curl Gujarat virus *(ToLCGV) to form infectious pseudorecombinant mixtures in both directions, might explain the reported synergistic effect of both tripartite mixtures. Our results suggest that the A component from both viruses are required to induce a synergism. In addition, they require the presence of at least one B component that is able to provide the movement functions to both A components.

Synergism has been explained in RNA virus mixtures by the presence of an efficient RNA silencing suppressor. The first, and probably most studied, example is the HC-Pro protein from members of the potyvirus family [31,32]. RNA silencing suppressors have also been reported in some begomoviruses [33,34]. In most cases, the activity has been associated to TrAP, a multifunctional protein originally described as activator of *CP *and *BR1 *(*NSP*) genes [30,35]. In ACMV-[CM] and *Sri-Lankan cassava mosaic virus *(SLCM) however, the *AC4 *genes have been also associated to RNA silencing [34].

In our system, the similarities with previously reported systems (increased symptom severity and viral DNA concentration) suggested that an RNA silencing suppressor activity is involved in the process. In an attempt to identify which viral genes are involved in this phenomenon, we carried out the inoculation experiments using mutant viruses. In the experiments with CP- mutants, synergism was observed in both cases (PepGMV + PHYVV CP- or PHYVV + PepGMV CP-) suggesting that both *CP *genes are not required for the observed synergism. As reported earlier for PYHVV, CP is not essential for infectivity in pepper plants [24]. This was also confirmed later when *CP *was replaced by a multiple cloning site to generate a VIGS vector [36], or by *GFP *gene to generate a tagged-virus [8]. Nevertheless, it is also possible that the CP from the wt virus 1 could complement the CP- version of the second virus as previously reported for the combination of PHYVV and *Tomato mottle Taino virus *(ToMoTV) [24]. Interestingly, the accumulation of viral DNA observed with CP- mutants tends to be lower when compared with the respective wt virus. Nevertheless, the symptom severity seems to be not affected.

In the experiments with mixed infections using the Rep mutant of virus 1 (either PHYVV or PepGMV) co-inoculated with the corresponding wt virus 2, no synergism was observed. These results are not unexpected since the differences in the respective iteron sequences from PHYVV and PepGMV predict an incompatibility for heterologous Rep binding [37]. Both Rep- mutants were not infective when singly inoculated in pepper plants.

Finally, we also inoculated pepper plants with mixtures in which one of the viruses was TrAP-. Interestingly, the mixture PHYVV TrAP- and wt PepGMV was able to induce synergism, whereas the opposite mixture, PepGMV TrAP- and wt PHYVV, did not. This suggested that in the case of PepGMV, TrAP might act as an RNA silencing suppressor as reported in other begomoviruses, and in its absence no synergism is observed. However, in the case of PHYVV, the RNA silencing suppressor activity might reside in another region/gene not in *TrAP*, although a weak suppressor activity of PHYVV TrAP cannot be discarded. PHYVV presents an *AC4*-like gene whose functionality has not been confirmed yet. The fact that PepGMV cannot complement, and therefore, move wt PHYVV A, eliminated the possibility of carrying out tripartite mixture inoculations with PHYVV TrAP- to corroborate that the suspected RNA silencing suppressor is located in PHYVV A.

One possibility for the asymmetric complementation of TrAP mutants can be suggested from the *in situ *hybridization experiments. The number of PepGMV infected cells is much higher than the number of PHYVV infected cells. Therefore, it is possible that the complementation can only work when both viruses are present in the same cell. In the double infections the number of infected cell with each virus increases, however, the number of PHYVV infected cells never reaches the numbers observed with PepGMV. The *in situ *hybridization experiments also suggested that even in the double infected plants, each virus follows its own program (low number of infected cells for PYHVV and high number for PepGMV). Thus, PepGMV mutants will have a low probability of complementation since it needs to coincide with a PHYVV-infected cell whose number is rather low. The opposite case also applies. PHYVV mutant has a better opportunity to be complemented since the probability of arriving to a PepGMV infected cell is higher due to its larger number of the cells containing PepGMV.

The relatively low probability of finding vascular tissue cells that are doubly infected suggests that the synergism is caused by an effect of the viral proteins on the plant metabolism (defenses) that facilitates virus replication rather than a direct interaction between proteins from both viruses with a host factor. This also agrees with the models that synergism in geminiviruses infected plants is due to an RNA silencing suppression mechanism. Mention should be given to the fact that in double infections both viruses are found in more host cells in leaf primordia, but also that cells apparently not infected in single infections now harbor these viruses. This could explain the increased concentration of both viruses in double infections, and this in turn could be caused by the induction of a host gene (or genes) that contributes to the movement and, perhaps, replication of both viruses in leaf primordia.

Previously, it was shown that in *in vitro *experiments, both TrAP proteins are able to transactivate the heterologous CP promoter, suggesting that a functional complementation is possible [30]. Therefore, the effect observed here with *in vivo* experiments might be a physical, localization problem due to the low probability of having doubly infected cells.

## Conclusions

In this report we have further characterized the synergism observed in pepper plants infected simultaneously with PepGMV and PHYVV. We have determined with quantitative PCR assays the concentration of both viruses increase in mixed infections. Interestingly PepGMV concentrations can be as much as two-order of magnitude higher than the concentration observed for PHYVV. Nevertheless, both viruses are localized to vascular tissue cells as shown through *in situ *hybridization experiments. In correlation with the enhanced DNA concentration, the number of infected cells also increased for both viruses in mixed infections. No changes in tissue tropism were observed in mixed infected plants; however, it should be mentioned that in this case both viruses infect cells in leaf primordia that do not normally harbor virus in single infections.

The results obtained using several mutants for both viruses suggest that both *CP *genes and PHYVV *TrAP *gene are not required for synergism. This also suggests that an RNA silencing suppression activity might reside, for PHYVV, in a different gene as reported for cassava infecting geminiviruses. Unfortunately, the lack of infectivity of PepGMV TrAP mutant made impossible to confirm the requirement of PepGMV TrAP for synergism.

Several observations suggest that each virus maintain its own replicative regulation since even in the doubly infected plants, where plants defenses are expected to be diminished, PHYVV does not reach the concentration and the number of infected cells observed with PepGMV. This might suggest that this self-regulation does not rely on RNA silencing mechanisms.

## Materials and methods

### Plant material

Pepper (*Capsicum annuum *L.) var. Sonora Anaheim seeds were germinated in a controlled environment chamber at 26 to 28°C with a 16:8 h (light/dark) photoperiod.

### Viral clones

Dimeric clones of PHYVV and PepGMV (Tamaulipas isolate) genomes used on this work have been previously described [19,38].

### Construction of PHYVV and PepGMV mutants

The following monomeric mutant clones were used in this work: PHYVVCP- (PHVCP191[24]); PHYVVTrAP- (TrAP mutant; [30]); PHYVVRep- (Shimada-Beltrán and Rivera-Bustamante, unpublished data). The PepGMV CP mutant was constructed introducing two new restriction sites in the CP open reading frames (ORF) by PCR using the forward primer F5'TG**ATTTAAAT**ATGGGGCCTAAATTC3' and the reverse primer R5'GG**ATTTAAAT**ATTAAACGCCATGGG3', which introduce *Swa*I restriction site. The PCR product was digested with *Swa*I and religated originating a deletion and frame change of CP. Additional PepGMV TrAP and Rep mutants (PepGMV TrAP- and PepGMV Rep-, respectively) were similarly constructed. The combination of primers F5'CC**ATTTAAAT**GGTCTATGCGTCGTC3' and R5'CT**ATTTAAAT**CTCC ACATCAACTGC3' were used to introduce *Swa*I restriction site in the TrAP ORF, whereas the primers F5'CG**ATTTAAAT**GTCCTTGGATGCCTG3' and R5'AGA**TTTAAAT**TATTGTGAATCTGGG3' were used to introduce similar sites in the Rep ORF. PCR products were amplified, enzyme restricted and religated originating a deletion and frame change of *TrAP *and *Rep*. The aforementioned modifications were confirmed by sequencing (Table 2).

**Table 2:** PHYVV and PepGMV viral mutant clones.

VIRUS	VIRAL MUTANT CLONE	MUTANT GENE	REFERENCE
PHYVV	PHYVV CP-	*CP*	Guevara-González *et al*., 1999

PHYVV	PHYVV TrAP-	*TrAP*	Ruiz-Medrano *et al*., 1999

PHYVV	PHYVV Rep-	*Rep*	Shimada-Beltrán and Rivera-Bustamante, unpublished data

PepGMV	PepGMV CP-	*CP*	This work

PepGMV	PepGMV TrAP-	*TrAP*	This work

PepGMV	PepGMV Rep-	*Rep*	This work

### Plant inoculation with DNA viral and tissue harvest

Plants at four-leaf stage were inoculated on third and fourth leaves with infectious clones through biolistic delivery with a low-pressure apparatus as described previously [19]. The following timeline was used for tissue collection: (i) first leaves level at 7 days post-inoculation (dpi), (ii) second leaves level at 14 dpi and (iii) third leaves level (21 dpi). All monomeric mutants were excised from the plasmid vector before inoculation. All dimeric clones were bombarded without restriction.

### Total DNA extraction

Total DNA from infected and mock-inoculated plants was extracted as described previously [8] and used for Southern blot analysis and viral quantification experiments.

### Southern blot analysis

Ten micrograms of total DNA per well was loaded in 1% agarose gel. After electrophoresis, DNA was transferred as previously described [19]. We used the intergenic region (IR) from PepGMV and IR from PHYVV as a probe [8]. Probes were radioactively labeled using Rediprime II kit and (α-^32^P) dCTP (Amersham). Hybridization was carried out at 65°C.

### Viral DNA quantification by real-time PCR

The real-time quantitative PCR procedures were previously described [19]. The primers used for PepGMV quantification was as follows: for *Rep *gene (encoding replication-associated protein), PepGMVRepq5' and PepGMVRepq3' [19]. The primers for internal normalization (designed for the 18 S RNA pepper sequence) were: 18Sq5'and 18Sq3' [19]. The primers used for PHYVV quantification, were TrAP/REn-For and TrAP/REn-Rev, previously described [39]. Relative units were calculated vs. the sample with the minor concentration.

### *In situ *hybridization

Leaves and meristematic tissues were excised from 7 dpi plants (first level of leaves) and processed as described by Ruiz-Medrano *et al*. (1999-b). Excised tissue was placed in paraffin blocks and sectioned with a Microm HM315 microtome (Walldorf, Germany). The intergenic region (IR) from PHYVV and PepGMV were cloned in PCRII TOPO (Invitrogen) and digested with *Bam*HI (PHYVV probe) or *Hind*III (PepGMV probe), as template. Generation of riboprobes was carried out by *in vitro *transcription (Promega) of IR from PHYVV and PepGMV, using digoxigenin (Dig)-labeled UTP (Roche). Sections were hybridized overnight on hybridization solution at 42°C in a moist chamber. Following the hybridization and high-stringency washed (wash solution: 2 × SSC/50% formamide), Dig-labeled RNA was detected using anti-Dig antibody (Roche) conjugated to alkaline phosphatase (AP). AP activity was determined using NBT-BCIP (Nitroblue tetrazolio/bromo-chlorine-indol phosphate; Roche). After the reaction sections were dehydrated and photographed with a digital camera adapted to a microscope for further analysis. The lack of cross-hybridization between PHYVV and PepGMV using these probes was corroborated previously using Southern blots.

## Competing interests

The authors declare that they have no competing interests.

## Authors' contributions

IRC carried out most experimental work and collaborated in writing the manuscript.

RFRB designed and coordinated the experiments and collaborated in writing the manuscript.

RRM and BXC helped in the design of experiments and writing the manuscript. All authors read and approved the final manuscript.

## References

[B1] Fauquet CM Briddon RW Brown JK Moriones E Stanley J Zerbini M Zhou X Geminivirus strain demarcation and nomenclature Arch Virology 2008 153 783 821 10.1007/s00705-008-0037-6 18256781

[B2] Torres-Pacheco I Garzon-Tiznado JA Brown JK Becerra-Flora A Rivera-Bustamante RF Detection and distribution of geminiviruses in Mexico and the southern United States Phytopathology 1996 86 1186 1192 10.1094/Phyto-86-1186

[B3] Torres-Pacheco I Garzón-Tiznado JA Herrera-Estrella L Rivera-Bustamante RF Complete nucleotide sequence of *Pepper huasteco virus*: Analysis and comparison with bipartite geminiviruses JGV 1993 74 2225 2231 10.1099/0022-1317-74-10-2225 8409944

[B4] Stenger DC Duffus JE Villalon B Biological and genomic properties of a geminivirus isolated from pepper Phytopathology 1990 80 704 709 10.1094/Phyto-80-704

[B5] Garzón-Tiznado JA Torres-Pacheco I Ascencio-Ibañez JT Herrera-Estrella L Rivera-Bustamante RF Inoculation of peppers with infectious clones of a new geminivirus by a biolistic procedure Phytopathology 1993 83 514 521

[B6] Méndez-Lozano J Rivera-Bustamante RF Fauquet CM De la Torre-Almaraz R *Pepper huasteco virus *and *Pepper golden mosaic virus *are geminiviruses affecting tomatillo (*Physalis ixocarpa*) crops in Mexico Plant Dis 2001 85 1291 10.1094/PDIS.2001.85.12.1291A 30831814

[B7] Ascencio-Ibañez JT Argüello-Astorga GR Méndez-Lozano J Rivera-Bustamante RF First report of Rhynchosia golden mosaic virus (RhGMV) infecting tobacco in Chiapas, Mexico Plant Dis 2002 86 692 10.1094/PDIS.2002.86.6.692C 30823249

[B8] Méndez-Lozano J Torres-Pacheco I Fauquet CM Rivera-Bustamante RF Interactions between geminiviruses in a naturally occurring mixture: Pepper huasteco virus and Pepper golden mosaic virus Phytopathology 2003 93 270 277 18944336 10.1094/PHYTO.2003.93.3.270

[B9] Zhou XP Liu YL Calvert L Munoz C OtimNape GW Robinson DJ Harrison BD Evidence that DNA-A of a geminivirus associated with severe cassava mosaic disease in Uganda has arisen by interspecific recombination JGV 1997 78 2101 2111 10.1099/0022-1317-78-8-2101 9267014

[B10] Harrison BD Zhou X Otim-Nape GW Liu Y Robinson DJ Role of a novel type of double infection in the geminivirus-induced epidemic of severe cassava mosaic in Uganda Annals of Applied Biology 1997 131 437 448 10.1111/j.1744-7348.1997.tb05171.x

[B11] Fondong VN Pita JS Rey ME de Kochko A Beachy RN Fauquet CM Evidence of synergism between African cassava mosaic virus and a new double-recombinant geminivirus infecting cassava in Cameroon JGV 2000 81 287 297 10.1099/0022-1317-81-1-287 10640569

[B12] Pita JS Fondong VN Sangare A Otim-Nape GW Ogwal S Fauquet CM Recombination, pseudorecombination and synergism of geminiviruses are determinant keys to the epidemic of severe cassava mosaic disease in Uganda JGV 2001 82 655 665 10.1099/0022-1317-82-3-655 11172108

[B13] Hou YM Gilbertson RL Increased pathogenicity in a pseudorecombinant bipartite geminivirus correlates with intermolecular recombination J Virol 1996 70 5430 5436 8764054 10.1128/jvi.70.8.5430-5436.1996 PMC190500

[B14] Hou YM Paplomatas EJ Gilbertson RL Host adaptation and replication properties of two bipartite geminiviruses and their pseudorecombinants Mol Plant-Microbe Interact 1998 11 208 217 10.1094/MPMI.1998.11.3.208

[B15] Vanitharani R Chellappan P Pita JS Fauquet CM Differential roles of AC2 and AC4 of cassava geminiviruses in mediating synergism and suppression of posttranscriptional gene silencing J Virol 2004 78 9487 9498 10.1128/JVI.78.17.9487-9498.2004 15308741 PMC506916

[B16] Morilla G Krenz B Jeske H Bejarano ER Wege C Tete a tete of tomato yellow leaf curl virus and Tomato yellow leaf curl Sardinia virus in single nuclei J Virol 2004 78 10715 10723 10.1128/JVI.78.19.10715-10723.2004 15367638 PMC516410

[B17] Chakraborty S Vanitharani R Chattopadhyay B Fauquet CM Supervirulent pseudorecombination and asymmetric synergism between genomic components of two distinct species of begomovirus associated with severe tomato leaf curl disease in India JGV 2008 89 818 828 10.1099/vir.0.82873-0 18272774

[B18] Alves M Alfenas-Zerbini P Andrade EC Esposito DA Silva FN da Cruz ACF Ventrella MC Otoni WC Zerbini FM Synergism and negative interference during co-infection of tomato and Nicotiana benthamiana with two bipartite begomoviruses Virology 2009 387 257 266 10.1016/j.virol.2009.01.046 19282016

[B19] Carrillo-Tripp J Lozoya-Gloria E Rivera-Bustamante RF Symptom remission and specific resistance of pepper plants after infection by *Pepper golden mosaic virus* Phytopathology 2007 97 51 57 10.1094/PHYTO-97-0051 18942936

[B20] Rodriguez-Negrete EA Carrillo-Tripp J Rivera-Bustamante RF RNA Silencing against Geminivirus: Complementary Action of Posttranscriptional Gene Silencing and Transcriptional Gene Silencing in Host Recovery J Virol 2009 83 1332 1340 10.1128/JVI.01474-08 19019951 PMC2620903

[B21] Morra MR Petty ITD Tissue specificity of geminivirus infection is genetically determined Plant Cell 2000 12 2259 2270 10.1105/tpc.12.11.2259 11090223 PMC150172

[B22] Wege C Saunders K Stanley J Jeske H Comparative analysis of tissue tropism of bipartite geminiviruses Journal of Phytopathology-Phytopathologische Zeitschrift 2001 149 359 368 10.1046/j.1439-0434.2001.00640.x

[B23] Qin Y Petty ITD Genetic analysis of bipartite geminivirus tissue tropism Virology 2001 291 311 323 10.1006/viro.2001.1205 11878900

[B24] Guevara-González RG Ramos PL Rivera-Bustamante RF Complementation of coat protein mutants of pepper huasteco geminivirus in transgenic tobacco plants Phytopathology 1999 89 540 545 18944688 10.1094/PHYTO.1999.89.7.540

[B25] Rothenstein D Krenz B Selchow O Jeske H Tissue and cell tropism of Indian cassava mosaic virus (ICMV) and its AV2 (precoat) gene product Virology 2007 359 137 145 10.1016/j.virol.2006.09.014 17049959

[B26] Wege C Pohl D Abutilon mosaic virus DNA B component supports mechanical virus transmission, but does not counteract begomoviral phloem limitation in transgenic plants Virology 2007 365 173 186 10.1016/j.virol.2007.03.041 17462695

[B27] Rasheed MS Selth L Koltunow A Randles J Rezaian M Single-stranded DNA of Tomato leaf curl virus accumulates in the cytoplasm of phloem cells Virology 2006 348 120 132 10.1016/j.virol.2005.11.054 16457866

[B28] Kim KS Shock TL Goodman RM Infection of *Phaseolus vulgaris *by bean golden mosaic virus: Ultrastructural aspects Virology 1978 89 22 33 10.1016/0042-6822(78)90036-3 685179

[B29] Sudarshana MR Wang HL Lucas WJ Gilbertson RL Dynamics of bean dwarf mosaic geminivirus cell-to-cell and long-distance movement in Phaseolus vulgaris revealed, using the green fluorescent protein Mol Plant-Microbe Interact 1998 11 277 291 10.1094/MPMI.1998.11.4.277

[B30] Ruiz-Medrano R Guevara-Gonzalez RG Arguello-Astorga GR Monsalve-Fonnegra Z Herrera-Estrella LR Rivera-Bustamante RF Identification of a sequence element involved in AC2-mediated transactivation of the *Pepper huasteco virus *coat protein gene Virology 1999 253 162 169 10.1006/viro.1998.9484 9918875

[B31] Vance VB Berger PH Carrington JC Hunt AG Shi XM 5' proximal potyviral sequence mediates potato virus X/potyviral synergistic disease in transgenic tobacco Virology 1995 206 583 590 10.1016/S0042-6822(95)80075-1 7831814

[B32] Shi X Miller H Verchot J Carrington J Vance V Mutations in the region encoding the central domain of helper component-proteinase (HC-Pro) eliminate potato virus X/potyviral synergism Virology 1997 231 35 42 10.1006/viro.1997.8488 9143300

[B33] Bisaro DM Silencing suppression by geminivirus proteins Virology 2006 344 158 168 10.1016/j.virol.2005.09.041 16364747

[B34] Vanitharani R Chellappan P Fauquet CM Geminiviruses and RNA silencing Trends Plant Sci 2005 10 144 151 15749473 10.1016/j.tplants.2005.01.005

[B35] Sunter G Bisaro DM Regulation of a geminivirus coat protein promoter by AL2 protein (TrAP): evidence for activation and derepression mechanisms Virology 1997 232 269 280 10.1006/viro.1997.8549 9191840

[B36] Abraham-Juarez MD Rocha-Granados MD Lopez MG Rivera-Bustamante RF Ochoa-Alejo N Virus-induced silencing of Comt, pAmt and Kas genes results in a reduction of capsaicinoid accumulation in chili pepper fruits Planta 2008 227 681 695 10.1007/s00425-007-0651-7 17999078

[B37] Argüello-Astorga GR Guevara-González RG Herrera-Estrella LR Rivera-Bustamante RF Geminivirus replication origins have a group-specific organization of iterative elements: A model for replication Virology 1994 203 90 100 8093156 10.1006/viro.1994.1458

[B38] Bonilla-Ramírez GM Guevara-González RG Garzon-Tiznado JA Ascencio-Iban˜ez JT Torres-Pacheco I Rivera-Bustamante RF Analysis of the infectivity of monomeric clones of pepper huasteco virus JGV 1997 78 947 951 10.1099/0022-1317-78-4-947 9129670

[B39] Shimada-Beltrán H Rivera-Bustamante RF Early and late gene expression in pepper huasteco yellow vein virus JGV 2007 88 3145 3153 10.1099/vir.0.83003-0 17947542

